# Anandamide in First-Episode Psychosis: Associations with Symptoms and Inflammation

**DOI:** 10.1192/j.eurpsy.2026.10539

**Published:** 2026-07-31

**Authors:** W. Ben Flah, R. Lansari, A. Kamoun, I. Ghazzani

**Affiliations:** Psychiatry, Faculty of Medicine Of Tunis, Tunis, Tunisia

## Abstract

**Introduction:**

Schizophrenia (SCZ) is a severe disorder with partially understood pathophysiology. The early stage is crucial for understanding pathogenic mechanisms and improving treatment strategies. Anandamide (AEA), a neurotransmitter of the endocannabinoid system, may be protective against psychosis.

**Objectives:**

This study aimed to measure serum AEA levels in first-episode psychosis (FEP) at admission (T0) and remission (T1), compare them to healthy controls and explore clinical and inflammatory correlates of AEA levels

**Methods:**

In a longitudinal observational study (Nov 2021–Dec 2023), 69 FEP patients and 74 controls underwent sociodemographic, clinical, psychometric assessment (PANSS, GAF, CGI, CAST) and blood tests (high sensitivity-CRP (hs-CRP), AEA, metabolic parameters).

**Results:**

Patients were predominantly young, single men, with high unemployment; one-third used cannabis. At T0, AEA levels were significantly higher in patients than in controls (p = 0.045) and remained stable at T1. AEA showed consistent negative correlations with all PANSS subscales (e.g., total PANSS at T0: r = −0.45, p<0.001; T1: r = −0.41, p=0.034),and CGI-severity scale (At T0: r = −0.316, p = 0.008). Its elevation was independent of cannabis use, and its association with FEP persisted after adjustment (OR = 3.54, 95% CI = 1.32–9.46; p = 0.012). A positive correlation with hs-CRP was observed at T0 (r = 0.24, p = 0.048), independent of other confounding factors such as smoking and metabolic parameters.

**Conclusions:**

AEA is elevated in FEP, inversely associated with symptom severity and positively correlated with inflammation, suggesting a potential neuroprotective and anti-inflammatory role. The endocannabinoid system may represent a promising target for novel therapeutic strategies.

**Disclosure of Interest:**

None Declared

